# Signal and Information Processing in Networks

**DOI:** 10.3390/e25121643

**Published:** 2023-12-11

**Authors:** Minyu Feng, Liang-Jian Deng, Feng Chen

**Affiliations:** 1 College of Artificial Intelligence, Southwest University, Chongqing 400715, China; 2 School of Mathematical Sciences, University of Electronic Science and Technology of China, Chengdu 611731, China

Networks are omnipresent in the realm of science, serving as a central focus in our modern world. As various networks such as digital networks, wireless sensor networks (WSNs), Internet of Things (IoTs), Internet of Vehicles (IoVs), brain networks (BNs), artificial neural networks (ANNs), and social networks (SNs) burgeon, there is a rapidly growing scarcity in handling the signals and information these networks generate. However, with the escalation of network complexity and entropy, the capability to analyze signals and information within diverse networks is at risk within the realm of information theory. The exponential growth of modern network complexity makes extracting meaningful insights from generated large amounts of data challenging, leading to a higher risk of losing valuable information. In network analysis, upgrading network complexity often leads to higher entropy. Ulteriorly, this makes it more difficult to distinguish related signals from noise, affecting the accuracy and reliability of information analysis. Addressing these challenges requires continuous research, innovation, and the development of robust analytical techniques in signals and information. Leveraging contemporary technologies such as distributed estimation, graph signal processing, graph neural networks, and deep learning could greatly aid in scrutinizing signals and information within these various networks. Moreover, modeling networks and analyzing their structural and dynamic behaviors can significantly contribute to the study of signal and information processing. Hence, signal and information processing within networks has emerged as a noteworthy domain within information theory.

In an era characterized by the substantial growth of information technology, the information industry has emerged as a vanguard, effecting a profound transformation in the manner in which we establish connections, facilitate communication, and share information. This industry has not only facilitated the convenience of researchers in their exploration, analysis, and utilization of information within networked environments, but has also catalyzed the development of innovative solutions to address a multitude of real-world challenges, thereby establishing the foundational underpinnings for myriad practical applications. As a result, many studies collectively contributing to a comprehensive understanding of distributed algorithms, neural network robustness, synchronization in complex systems, community detection criteria, optimization over networks, and so on have been published and proposed in recent years, which have enhanced the field of signal processing and neural networks.

Concretely, Qing (contribution 1) addresses the challenge of comparing community detection methods across different network models. They introduced the four-step criterion SCSTC, utilizing a separation condition and sharp threshold concepts. Their study reveals inconsistencies in community detection, particularly with the SPACL algorithm, prompting improvements in theoretical convergence rates using row-wise eigenvector deviation techniques. Zhang, Li, and Yang (contribution 2) systematically explore MAC scheduling strategies. Emphasizing both throughput and delay aspects, this study evaluates and compares their performance through theoretical analysis and simulations. The work provides a theoretical foundation for enhancing MAC scheduling technology in future wireless networks, acknowledging the significance of both throughput and delay in practical communication networks. In addition, Feng et al. (contribution 3) tackle nonsmooth composite optimization problems over networks with smooth and nonsmooth components, incorporating equality and box constraints for decision variables. Utilizing a multi-agent network approach, problems are decentralized; the paper establishes the Lagrange function, deriving first-order optimal conditions. It proposes a decentralized algorithm with proximal operators, showcasing uncoordinated stepsizes for agents or edges. Moreover, the work of Liu (contribution 5) introduces a distributed support vector ordinal regression (SVOR) algorithm. This method avoids transmitting original data through random feature maps and mitigates high approximation dimensions through sparse regularization. The research of Cohen et al. (contribution 8) explores the impact of dropout regularization on neural network robustness against adversarial attacks. It also introduces the concept of “functional smearing” and finds that lower functional diversity enhances network robustness. (contribution 11) delves into the nonlinear aspects of projective synchronization within coupled memristive neural networks. The study addresses parameter mismatches, achieving synchronization through nonlinear control methods. Sufficient criteria for projective convergence are established under various projective factors and the Lyapunov–Krasovskii functional (LKF) framework.

Open-source information has become an important trend in this era; it can facilitate the rapid spread and wide exchange of information. As Xiaodong Zhang et al. point out (contribution 5), as a physical carrier for the implementation of open-source modes and ideas, the open-source community (OSC) has accumulated many volunteers from all over the world. However, the OSC’s characteristics (openness, knowledge, democracy, and collaboration) suggest that rigid organizational structures or strong control would hinder community creativity, potentially resulting in user loss and OSP failure. Therefore, it is significant to study the robustness of OSCs. Accordingly, Xiaodong Zhang et al. construct a directed, weighted, semantic-based multi-project knowledge collaboration network based on the characteristics of knowledge behavior and the semantic content generated by open-source projects. From the perspective of user attrition and behavioral degradation, two failure modes are constructed: node failure and edge failure. Based on empirical data from the Local Motors open-source vehicle design community, dynamic robustness analyses were conducted. Their findings indicate that the network they built displays varying levels of robustness in response to different failure modes and types of nodes. These findings can be used to provide a more comprehensive and targeted management reference, promoting the efficient development of OSCs.

The Internet of Things (IoT) is a rapidly advancing technological paradigm with applications in agriculture, smart buildings, manufacturing, and healthcare. However, the increasing demand for IoT network applications raises security concerns, particularly in identification and authentication. Gao (contribution 10) proposed radio frequency fingerprinting techniques to address these concerns, utilizing regular radio traffic for more secure identification and overcoming the limitations of traditional methods.

In the realm of secure communication, Han, Cheng, and Wei et al. (contribution 12) employed chaotic sequences for signal encryption and decryption, incorporating projective synchronization to enhance efficiency. The study introduced an adaptive control mechanism to improve signal decryption and decryption efficiency in secure communication. Addressing adversarial attacks in deep learning, Sardar, Khan, and Hintze et al. (contribution 9) explored the impact of dropout regularization on network resistance. Their findings indicate enhanced robustness within a specific range of dropout probabilities, particularly against fooling attacks. They suggested that dropout improves robustness to fooling. Ya-Ru Fan (contribution 6) integrated superpixel segmentation with principal component analysis for robust hyperspectral image (HSI) segmentation, effectively removing mixed noise. This approach, which combines segmentation techniques with principal component analysis, enhances the low-rank properties of HIS, including sparse noise and Gaussian noise.

Many of the above applications use AI algorithms, such as neural networks, deep learning, and machine learning. Problems such as overfitting, underfitting, gradient vanishing and gradient exploding occur in AI algorithm training. Many basic problems have mature solutions, but there are some special problems. Few-shot class incremental learning (FSCIL) is an extremely challenging but valuable problem in real-world applications. In the context of encountering new few-shot tasks at each incremental stage, the simultaneous challenges of retaining previously learned knowledge while preventing overfitting to new categories with limited training data must be considered. The problem of catastrophic forgetting in incremental learning is primarily caused by the drift of parameters in the model.

In few-shot class incremental learning (FSCIL), Zhang and Gu (contribution 7) proposed an efficient prototype replay and calibration (EPRC) method to address the challenge of overfitting. This approach involves saving prototype features for each encountered category and performing prototype replay and calibration during each few-shot incremental stage; this process improves the generalization capabilities of both the feature extractor and projection layer, effectively mitigating overfitting issues in few-shot learning.

With the rapid progress of manufacturing technology, the structure of mechanical equipment is becoming more and more complex, and the operating conditions of parts are becoming more and more severe. Accurate prediction of the health state of parts has become an important problem. Liu et al. (contribution 13) focused on predicting mechanical equipment health using multi-domain features and temporal convolutional networks. The study proposed a prediction method based on multi-domain features, using temporal convolutional networks for constructing a performance degradation trend prediction model.

In the field of medical research, information technology has also played a major role. Ma (contribution 14) utilized Granger causality with a polynomial kernel for magnetoencephalogram (MEG) signal analysis. This approach effectively distinguished between healthy individuals and those with depression based on the analysis of functional brain networks. Yoav et al. (contribution 15) proposed an automated deep learning algorithm for identifying cyclic alternating pattern (CAP) phases in EEG signals, contributing to sleep quality assessment. The algorithm integrates EEG signal contextual information and employs data augmentation methods to preserve the time–frequency structure.

In the era of thriving information technology, the information industry is a transformative force, reshaping how we connect, communicate, and share information. Serving as a catalyst for innovative solutions to real-world challenges, this industry supports researchers in exploring, analyzing, and applying information in networked environments. Countless recent studies have advanced our understanding of distributed algorithms, neural network robustness, synchronization in complex systems, and optimization over networks. These contributions significantly enrich the realms of signal processing and neural networks. Specifically, the research addresses challenges in community detection, MAC scheduling, nonsmooth composite optimization problems, distributed support vector ordinal regression (SVOR), neural network robustness against adversarial attacks, and nonlinear aspects of projective synchronization within coupled memristive neural networks. Furthermore, the significance of open-source information and the robustness of open-source communities (OSCs) are emphasized, shedding light on their dynamics and potential failures.

In the field of the Internet of Things (IoT), the lack of adequate identification and authentication is recognized as a significant security concern. Innovative solutions such as radio frequency fingerprinting techniques are proposed to enhance identification security. Additionally, encryption and decryption technologies, anti-attack technology, and data recovery technology are highlighted for safeguarding information security. Deep learning tasks face adversarial attacks, and the impact of dropout regularization on neural networks is explored. The study suggests that dropout regularization can enhance network resistance to adversarial attacks within a specific range of dropout probabilities, emphasizing the need for further research on complex datasets, networks, and training regimes. Applications in areas such as medicine and manufacturing are discussed, including the prediction of mechanical equipment health using multi-domain features and temporal convolutional networks, and the construction of functional brain networks for diagnosing and treating mental illness.

In summary, we have delved into the processing of signals and information within networks, an indispensable component in the realm of science. With the rapid development of various networks such as digital networks, wireless sensor networks (WSNs), Internet of Things (IoTs), Internet of Vehicles (IoVs), brain networks (BNs), artificial neural networks (ANNs), and social networks (SNs), there is a growing challenge in handling the signals and information generated by these networks. However, as network complexity and entropy escalate, the capability to analyze signals and information within diverse networks is at risk in the field of information theory.

Looking ahead, there is a promising landscape for research and advancements in signal and information processing within networks, driven by the continuous emergence of new technologies and applications. The new potential of distributed sensing and data or information sources in networks deserves further development. Joint communications and sensing in networks necessitate the development of new models and algorithms for efficient information processing. Ongoing studies in various areas, such as network security, the Internet of Things (IoT), deep learning, and other relevant fields, will contribute significantly to information processing. For instance, advancements in network security will enhance the protection of sensitive information and ensure the integrity of the data being processed. The IoT will continue to expand the networked devices and sensors, generating extensive data that can be leveraged for insightful analysis. Deep learning techniques will enable more sophisticated and accurate analysis of signals and information, unlocking new possibilities for network intelligence. Moreover, the continuous evolution of technology and the development of novel applications will drive the need for innovative models and algorithms for information processing. Researchers will focus on developing adaptive and scalable approaches that can handle the dynamic nature of networks and the diverse data or information sources involved. These advancements will ameliorate the accuracy and efficiency of information processing, improve the capability to analyze signals and information, and significantly address the risk of the capability to analyze signals and information within diverse networks within the realm of information theory.

